# A Review of Intraocular Biomolecules in Retinal Vein Occlusion: Toward Potential Biomarkers for Companion Diagnostics

**DOI:** 10.3389/fphar.2022.859951

**Published:** 2022-04-26

**Authors:** Bingjie Wang, Xiao Zhang, Huan Chen, Adrian Koh, Chan Zhao, Youxin Chen

**Affiliations:** ^1^ Department of Ophthalmology, Peking Union Medical College Hospital, Chinese Academy of Medical Sciences, Beijing, China; ^2^ School of Medicine, Tsinghua University, Beijing, China; ^3^ Key Laboratory of Ocular Fundus Diseases, Chinese Academy of Medical Sciences and Peking Union Medical College, Beijing, China; ^4^ Eye & Retina Surgeons, Camden Medical Centre, Singapore, Singapore

**Keywords:** biomarker, retinal vein occlusion, aqueous humor, companion diagnostic, cytokine

## Abstract

Retinal vein occlusion (RVO) is one of the most common retinal vascular diseases. The pathogenesis of RVO is multifactorial and involves a complex interplay among a variety of vascular and inflammatory mediators. Many cytokines, chemokines, growth factors, and cell adhesion molecules have been reported to be implicated. Treatments for RVO are directed at the management of underlying risk factors and vision-threatening complications, including macula edema (ME) and neovascularization. Intravitreal anti-VEGF agents are currently considered as the first-line treatment for ME secondary to RVO (RVO-ME), but a substantial proportion of patients responded insufficiently to anti-VEGF agents. Since RVO-ME refractory to anti-VEGF agents generally responds to corticosteroids and its visual outcome is negatively correlated to disease duration, prediction of treatment response at baseline in RVO-ME may significantly improve both cost-effectiveness and visual prognosis. Several bioactive molecules in the aqueous humor were found to be associated with disease status in RVO. This review aims to present a comprehensive review of intraocular biomolecules reported in RVO, including VEGF, IL-6, IL-8, MCP-1, sICAM-1, IL-12, IL-13, sVEGFR-1, sVEGFR-2, PDGF-AA, etc., highlighting their association with disease severity and/or phenotype, and their potential roles in prognostic prediction and treatment selection. Some of these molecules may serve as biomarkers for aqueous humor-based companion diagnostics for the treatment of RVO in the future.

## Introduction

Retinal vein occlusion (RVO) is one of the most common retinal vascular diseases (Branch Vein Occlusion Study Group, 1986; The Central Vein Occlusion Study, 1993). It is caused by partial or complete occlusion of venous blood flow, which leads to an increase in venous pressure with subsequent leakage of the retinal microvasculature proximal to the occlusion site (Christoffersen and Larsen, 1999). Blockage of the main retinal vein is called central retinal vein occlusion (CRVO), and of a smaller vein is called branch retinal vein occlusion (BRVO). The estimated 15-year cumulative incidence of RVO was reported to be 2.3% in the population, with BRVO and CRVO representing 1.8 and 0.5%, respectively (Klein et al., 2008). In a meta-analysis that pooled data from the United States, Europe, Asia, and Australia, about 16.4 million people were affected by RVO worldwide in 2008 (Rogers et al., 2010). The pathogenesis of RVO is multifactorial and involves a complex interplay among a variety of vascular and inflammatory mediators. While vascular endothelial growth factor (VEGF), a potent mediator of both vascular permeability and inflammation, undoubtedly plays a central role in the pathological process of RVO, several cytokines, chemokines, growth factors, and cell adhesion molecules have been reported to be implicated (Noma et al., 2019; 2020).

Treatments for RVO are directed at the management of underlying risk factors and vision-threatening complications, including macula edema (ME) and neovascularization. Intravitreal anti-VEGF agents are currently considered as the first-line treatment for ME secondary to RVO (RVO-ME) (Schmidt-Erfurth et al., 2019), but a substantial proportion of patients responded insufficiently to anti-VEGF agents. Since RVO-ME refractory to anti-VEGF agents generally responds to corticosteroids and its visual outcome is negatively correlated to disease duration (Wallsh and Gallemore, 2021), prediction of treatment response at base line in RVO-ME may significantly improve both cost-effectiveness and visual prognosis.

A companion diagnostic is a set of diagnostic tests that predict the safety and/or effectiveness of a particular treatment and has been increasingly recognized as a means to improve the precision of treatments in cancer (Rosenbaum and Weisman, 2017). Several bioactive molecules in the aqueous humor were found to be associated with disease status in RVO, and thus may serve as biomarkers for treatment prediction. In fact, customized intravitreal injections based on aqueous humor cytokines were proved to be beneficial in an intractable RVO-ME patient (Modi et al., 2021), and “liquid biopsy”, a close concept of companion dialogistic, was proposed to dictate treatments in diabetic retinopathy (Vujosevic and Simó, 2017). This review aims to present a comprehensive review of intraocular biomolecules reported in RVO, highlighting their association with disease severity and/or phenotype and their potential roles in prognostic prediction and treatment selection. The most studied intraocular biomolecules are listed in Table 1 and Table 2, and the least studied biomolecules are listed in Table 3.

**TABLE 1:** The most studied intraocular biomolecules associated with disease severity in RVO.

Biomolecules	Number of Studies Revealed Disease Association/Total Number of Studies	References
VEGF	44/48 revealed PR	(Rezar-Dreindl et al., 2017)^a^, (Noma et al., 2011e)^a^, (Noma et al., 2011a)^a^, (Noma et al., 2010c)^a^, (Noma et al., 2010b)^a^, (Noma et al., 2009)^a^, (Noma et al., 2008)^a^, (Noma et al., 2012b)^a^, (Noma et al., 2016b)^a^, (Kim et al., 2016)^a^, (Noma et al., 2012d)^a^, (Lim, 2011)^a^, (Noma et al., 2010a)^a^, (Noma et al., 2006)^a^, (Noma et al., 2005)^a^, (Boyd et al., 2002)^a^, (Noma et al., 2012e)^a^, (Park et al., 2010)^a^, (Mashima et al., 2019)^a^, (Noma et al., 2017), (Noma et al., 2013c)^a^, (Noma et al., 2014b)^a^, (Shchuko et al., 2015)^a^, (Park and Ahn, 2009)^a^, (Park and Ahn, 2008)^a^, (Noma et al., 2014c)^a^, (Jung et al., 2014)^a^, (Feng et al., 2013), (Noma et al., 2014a)^a^, (Funatsu et al., 2012)^a^, (Kaneda et al., 2011)^a^, (Noma et al., 2015)^a^, (Ki et al., 2007)^a^, (Noma et al., 2011d)^a^, (Okunuki et al., 2011)^a^, (Matsushima et al., 2019)^a^, (Noma et al., 2013b)^a^, (Noma et al., 2011b)^a^, (Noma et al., 2011c)^a^, (Noma et al., 2010d), (Dacheva et al., 2016)^a^, (Ehlken et al., 2011)^a^, (Sin et al., 2013), (Noma et al., 2012c)^a^, (Machalinska et al., 2016)^a^, (Bertelmann et al., 2014)^a^, (Tuuminen and Loukovaara, 2014b)^a^, (Yasuda et al., 2014)^a^
IL-6	29/33 revealed PR	(Rezar-Dreindl et al., 2017)^a^, (Campochiaro et al., 2009), (Noma et al., 2010c)^a^, (Noma et al., 2010b)^a^, (Noma et al., 2009)^a^, (Noma et al., 2008)^a^, (Noma et al., 2012b)^a^, (Noma et al., 2016b)^a^, (Lim, 2011)^a^, (Noma et al., 2006)^a^, (Noma et al., 2005)^a^, (Mashima et al., 2019)^a^, (Noma et al., 2017)^a^, (Noma et al., 2013c)^a^, (Noma et al., 2014b)^a^, (Funk et al., 2009)^a^, (Shchuko et al., 2015)^a^, (Park and Ahn, 2008)^a^, (Noma et al., 2014c)^a^, (Jung et al., 2014)^a^, (Feng et al., 2013)^a^, (Noma et al., 2014a)^a^, (Koss et al., 2012)^a^, (Funatsu et al., 2012)^a^, (Kaneda et al., 2011)^a^, (Noma et al., 2015)^a^, (Ki et al., 2007)^a^, (Chen et al., 1999)^a^, (Lee et al., 2012), (Noma et al., 2011d)^a^, (Noma et al., 2013b)^a^, (Zeng et al., 2019), (Dacheva et al., 2016)
IL-8	16/19 revealed PR	(Rezar-Dreindl et al., 2017)^a^, (Noma et al., 2016b)^a^, (Lim, 2011)^a^, (Mashima et al., 2019)^a^, (Noma et al., 2017)^a^, (Funk et al., 2009)^a^, (Shchuko et al., 2015)^a^, (Noma et al., 2014c)^a^, (Jung et al., 2014)^a^, (Feng et al., 2013), (Kaneda et al., 2011)^a^, (Noma et al., 2015)^a^, (Ki et al., 2007)^a^, (Lee et al., 2012)^a^, (Noma et al., 2011d)^a^, (Okunuki et al., 2011)^a^, (Zeng et al., 2019), (Dacheva et al., 2016), (Fonollosa et al., 2010)^a^
MCP-1	15/18 revealed PR	(Rezar-Dreindl et al., 2017)^a^, (Noma et al., 2016b)^a^, (Lim, 2011)^a^, (Mashima et al., 2019)^a^, (Noma et al., 2017)^a^, (Noma et al., 2014b)^a^, (Funk et al., 2009)^a^, (Shchuko et al., 2015)^a^, (Noma et al., 2014c)^a^, (Jung et al., 2014)^a^, (Noma et al., 2014a)^a^, (Kaneda et al., 2011)^a^, (Noma et al., 2015)^a^, (Noma et al., 2011d)^a^, (Okunuki et al., 2011), (Noma et al., 2013b)^a^, (Kunikata et al., 2012), (Dacheva et al., 2016)
sICAM-1	15/17 revealed PR	(Noma et al., 2011a)^a^, (Noma et al., 2016b)^a^, (Noma et al., 2012d)^a^, (Noma et al., 2012e)^a^, (Mashima et al., 2019)^a^, (Noma et al., 2017)^a^, (Noma et al., 2013c)^a^, (Noma et al., 2014b)^a^, (Noma et al., 2016d), (Noma et al., 2014c)^a^, (Noma et al., 2014a)^a^, (Noma et al., 2015), (Noma et al., 2011d)^a^, (Noma et al., 2013b)^a^, (Noma et al., 2011b)^a^, (Noma et al., 2011c)^a^, (Noma et al., 2010d)^a^
sVEGFR-2	7/12 revealed PR	(Noma et al., 2016b)^a^, (Mashima et al., 2019), (Noma et al., 2017), (Noma et al., 2014b)^a^, (Noma et al., 2014c)^a^, (Noma et al., 2014a), (Noma et al., 2015), (Noma et al., 2011d)^a^ (Noma et al., 2013b)^a^, (Noma et al., 2011b)^a^ (Noma and Mimura, 2013)^a^, (Noma et al., 2012c)^a^
PDGF-AA	7/11 revealed PR	(Rezar-Dreindl et al., 2017), (Noma et al., 2016b)^a^, (Lim, 2011)^a^, (Mashima et al., 2019)^a^, (Noma et al., 2017), (Funk et al., 2009), (Noma et al., 2014c)^a^, (Jung et al., 2014)^a^, (Noma et al., 2015), (Lee et al., 2012), (Noma et al., 2011d)^a^
IL-12	4/9 revealed NR, 1/9 revealed PR	(Rezar-Dreindl et al., 2017)^b^, (Mashima et al., 2019), (Noma et al., 2017), (Shchuko et al., 2015)^a^, (Noma et al., 2014c)^b^, (Kaneda et al., 2011), (Noma et al., 2015), (Ki et al., 2007), (Noma et al., 2011d)^b^
IL-13	2/7 revealed NR, 1/7 revealed PR	(Mashima et al., 2019), (Noma et al., 2017), (Shchuko et al., 2015)^a^, (Noma et al., 2014c)^b^, (Kaneda et al., 2011), (Noma et al., 2015), (Noma et al., 2011d)^b^
sVEGFR-1	6/6 revealed PR	(Noma et al., 2016b)^a^, (Mashima et al., 2019)^a^, (Noma et al., 2017)^a^, (Noma et al., 2014c)^a^, (Noma et al., 2015)^a^, (Noma et al., 2011d)^a^

PR: positively related to disease severity; NR: negatively related to disease severity.

a Studies revealed a positive correlation between the intraocular level of the biomolecule and disease severity.

b Studies revealed a negative correlation between the intraocular level of the biomolecule and disease severity.

Abbreviations: VEGF, vascular endothelial growth factor; IL, interleukin; MCP-1, monocyte chemoattractant protein -1; sICAM-1, soluble intercellular adhesion molecule-1; sVEGFR, Soluble VEGF receptors; PDGF, platelet-derived growth factor.

**TABLE 2:** Studies pertaining to intraocular biomolecules and treatments in RVO^a^.

Biomolecules	Number of Studies	References
VEGF	13	(Rezar-Dreindl et al., 2017; Campochiaro et al., 2009), (Mashima et al., 2019), (Noma et al., 2017), (Funk et al., 2009), (Shchuko et al., 2015), (Noma et al., 2016c), (Noma et al., 2016d), (Noma et al., 2016a), (Noma et al., 2021), (Sohn et al., 2014), (Matsushima et al., 2019), (Noma et al., 2013a)
IL-8	13	(Rezar-Dreindl et al., 2017), (Mashima et al., 2019), (Noma et al., 2017), (Funk et al., 2009), (Shchuko et al., 2015), (Noma et al., 2016c), (Noma et al., 2016d), (Noma et al., 2016a), (Noma et al., 2021), (Sohn et al., 2014), (Okunuki et al., 2011), (Matsushima et al., 2019), (Zeng et al., 2019)
IL-6	12	(Rezar-Dreindl et al., 2017), (Campochiaro et al., 2009), (Mashima et al., 2019), (Noma et al., 2017), (Funk et al., 2009), (Shchuko et al., 2015), (Noma et al., 2016c), (Noma et al., 2016d), (Noma et al., 2016a), (Noma et al., 2021), (Sohn et al., 2014), (Matsushima et al., 2019)
MCP-1	10	(Rezar-Dreindl et al., 2017), (Mashima et al., 2019), (Noma et al., 2017), (Shchuko et al., 2015), (Noma et al., 2016c), (Noma et al., 2016d), (Noma et al., 2016a), (Noma et al., 2021), (Matsushima et al., 2019), (Kunikata et al., 2012)
sICAM-1	8	(Mashima et al., 2019), (Noma et al., 2017), (Noma et al., 2016c), (Noma et al., 2016d), (Noma et al., 2016a), (Noma et al., 2021), (Matsushima et al., 2019), (Noma et al., 2013a)
IL-12	8	(Mashima et al., 2019), (Noma et al., 2017), (Shchuko et al., 2015), (Noma et al., 2016c), (Noma et al., 2016d), (Noma et al., 2016a), (Noma et al., 2021), (Kaneda et al., 2011)
sVEGFR-2	7	(Mashima et al., 2019), (Noma et al., 2017), (Noma et al., 2016c), (Noma et al., 2016d), (Noma et al., 2016a), (Noma et al., 2021), (Matsushima et al., 2019)
IL-13	7	(Mashima et al., 2019), (Noma et al., 2017), (Shchuko et al., 2015), (Noma et al., 2016c), (Noma et al., 2016d), (Noma et al., 2016a), (Noma et al., 2021)
sVEGFR-1	7	(Mashima et al., 2019), (Noma et al., 2017), (Noma et al., 2016c), (Noma et al., 2016d), (Noma et al., 2016a), (Noma et al., 2021), (Matsushima et al., 2019)
PDGF-AA	6	(Noma et al., 2016c), (Noma et al., 2016d), (Noma et al., 2016a), (Noma et al., 2021), (Sohn et al., 2014), (Matsushima et al., 2019)

a Studies which described changes of intraocular biomolecules in response to treatment or discussed associations between intraocular biomolecules and treatment response/disease recurrence are included in this table.

Abbreviations: VEGF, vascular endothelial growth factor; IL, interleukin; MCP, monocyte chemoattractant protein; sICAM, soluble intercellular adhesion molecule; sVEGFR, soluble VEGF receptors; PDGF, platelet-derived growth factor.

**TABLE 3:** Other intraocular biomolecules studied in RVO^a^.

Biomolecules	Number of Studies	References
PEDF	12	(Noma et al., 2011e), (Noma et al., 2012d), (Noma et al., 2010a), (Noma et al., 2012e), (Park et al., 2010), (Park and Ahn, 2009), (Noma et al., 2014a), (Noma et al., 2013b), (Noma et al., 2011c), (Noma et al., 2013a), (Dacheva et al., 2016), (Noma et al., 2012a)
PlGF	12	(Noma et al., 2016b), (Boyd et al., 2002), (Mashima et al., 2019), (Noma et al., 2017), (Noma et al., 2016c), (Noma et al., 2016d), (Noma et al., 2016a), (Noma et al., 2021), (Noma et al., 2014c), (Noma et al., 2015), (Noma et al., 2011d), (Matsushima et al., 2019)
TNF-α	11	(Rezar-Dreindl et al., 2017), (Campochiaro et al., 2009), (Lim, 2011), (Funk et al., 2009), (Shchuko et al., 2015), (Sohn et al., 2014), (Jung et al., 2014), (Kaneda et al., 2011), (Ki et al., 2007), (Chen et al., 1999), (Zeng et al., 2019)
IL-1β	9	(Rezar-Dreindl et al., 2017), (Campochiaro et al., 2009), (Funk et al., 2009), (Shchuko et al., 2015), (Sohn et al., 2014), (Feng et al., 2013), (Kaneda et al., 2011), (Ki et al., 2007), (Zeng et al., 2019)
IL-10	8	(Rezar-Dreindl et al., 2017), (Lim, 2011), (Funk et al., 2009), (Shchuko et al., 2015), (Sohn et al., 2014), (Kaneda et al., 2011), (Ki et al., 2007), (Zeng et al., 2019)
IL-2	8	(Rezar-Dreindl et al., 2017), (Funk et al., 2009), (Shchuko et al., 2015), (Sohn et al., 2014), (Kaneda et al., 2011), (Ki et al., 2007), (Chen et al., 1999), (Lee et al., 2012)
IFN-γ	6	(Lim, 2011), (Funk et al., 2009), (Shchuko et al., 2015), (Kaneda et al., 2011), (Lee et al., 2012), (Zeng et al., 2019)
IL-4	6	(Rezar-Dreindl et al., 2017), (Funk et al., 2009), (Shchuko et al., 2015), (Sohn et al., 2014), (Kaneda et al., 2011), (Zeng et al., 2019)
IL-5	6	(Rezar-Dreindl et al., 2017), (Funk et al., 2009), (Shchuko et al., 2015), (Sohn et al., 2014), (Kaneda et al., 2011), (Lee et al., 2012)
IP-10	6	(Rezar-Dreindl et al., 2017), (Funk et al., 2009), (Shchuko et al., 2015), (Sohn et al., 2014), (Jung et al., 2014), (Okunuki et al., 2011)
IL-15	5	(Rezar-Dreindl et al., 2017), (Funk et al., 2009), (Shchuko et al., 2015), (Sohn et al., 2014), (Kaneda et al., 2011)
IL-1α	5	(Rezar-Dreindl et al., 2017), (Funk et al., 2009), (Sohn et al., 2014), (Jung et al., 2014), (Kaneda et al., 2011)
MIP-1α	5	(Rezar-Dreindl et al., 2017), (Funk et al., 2009), (Shchuko et al., 2015), (Lee et al., 2012), (Kunikata et al., 2012)
bFGF	5	(Rezar-Dreindl et al., 2017), (Boyd et al., 2002), (Shchuko et al., 2015), (Feng et al., 2013), (Okunuki et al., 2011)
RANTES	4	(Funk et al., 2009), (Shchuko et al., 2015), (Okunuki et al., 2011), (Kunikata et al., 2012)
IL-17	4	(Rezar-Dreindl et al., 2017), (Shchuko et al., 2015), (Sohn et al., 2014), (Kaneda et al., 2011)
TGF-β	3	(Rezar-Dreindl et al., 2017), (Feng et al., 2013), (Tuuminen and Loukovaara, 2014a)
eotaxin	3	(Funk et al., 2009), (Shchuko et al., 2015), (Kunikata et al., 2012)
EPO	3	(Inomata et al., 2004), (Stahl et al., 2010), (Shin et al., 2014)
GM-CSF	3	(Funk et al., 2009), (Shchuko et al., 2015), (Okunuki et al., 2011)
IL-7	3	(Funk et al., 2009), (Shchuko et al., 2015), (Dacheva et al., 2016)
MIP-1β	3	(Rezar-Dreindl et al., 2017), (Shchuko et al., 2015), (Kunikata et al., 2012)
NO	2	(Fard and Dehpour, 2010), (Fard et al., 2010)
FGF-2	2	(Funk et al., 2009), (Lee et al., 2012)
PTX3	2	(Noma et al., 2014a), (Noma et al., 2013b)
VEGF165b	2	(Ehlken et al., 2011), (Baba et al., 2014)
Ang-1	2	(Dacheva et al., 2016), (Tuuminen and Loukovaara, 2014b)
Ang-2	2	(Rezar-Dreindl et al., 2017), (Tuuminen and Loukovaara, 2014b)

a Other biomolecules described in at least 2 independent studies are presented in this table.

Abbreviations: PEDF, pigment epithelium-derived factor; PlGF, placental growth factor; TNF, tumor necrosis factor; IL, interleukin; IFN, interferon; IP, IFN-γ induced protein; MIP, macrophage inflammatory protein; bFGF, basic fibroblast growth factor; RANTES, Regulated upon Activation, Normal T Cell Expressed and Presumably Secreted; TGF, transforming growth factor; GM-CSF, granulocyte macrophage colony-stimulating factor; NO, nitro oxide; FGF, fibroblast growth factor; PTX3, pentraxin 3; VEGF, vascular endothelial growth factor; Ang, angiopoietin; EPO, erythropoietin.

## Intraocular Biomarkers

### VEGF

In humans, the VEGF family includes VEGF-A (commonly referred to simply as VEGF), -B, -C, -D, and placental growth factor (PlGF). VEGF is an endothelial-cell-specific mitogen that promotes vascular permeability and angiogenesis (Keck et al., 1989). It is believed to be induced by the ischemic condition resulting from occlusion of retinal veins and plays an important role in RVO associated pathophysiological processes, including ME, the major cause of visual impairment, as well as neovascularization of the retina, optic disc, or the anterior segment, which may lead to vitreous hemorrhage or neovascular glaucoma (Chan et al., 2011). Intravitreal injection of anti-VEGF agents is, *de facto,* the most important treatment modality for RVO-ME. Intraocular VEGF levels are well demonstrated to be associated with disease severity in RVO from different aspects. VEGF concentrations in intraocular fluids were higher in CRVO than in BRVO (Campochiaro et al., 2009; Rezar-Dreindl et al., 2017), in ischemic than in nonischemic CRVO (Noma et al., 2008; Noma et al., 2009; 2010b; Noma et al., 2010c; Noma et al., 2011a; Noma et al., 2011e; Noma et al., 2012b), and in major BRVO than in macular BRVO (Lim, 2011; Noma et al., 2012d; Kim et al., 2016; Noma et al., 2016b). They were reported to be associated with severity of ME (Noma et al., 2005; Noma et al., 2006; Noma et al., 2008; Noma et al., 2009; Noma et al., 2010a; Noma et al., 2010b; Noma et al., 2010c; Noma et al., 2011a; Noma et al., 2011e), neovascularization of the iris (NVI) (Boyd et al., 2002; Noma et al., 2005; Noma et al., 2006; Noma et al., 2010a), serous retinal detachment (SRD) (Park et al., 2010; Noma et al., 2012e), electroretinogram parameters (Yasuda et al., 2011) and aqueous flare levels (Noma et al., 2013c; Noma et al., 2014b; Noma et al., 2017; Mashima et al., 2019). Intraocular VEGF level usually drop dramatically after an intravitreal anti-VEGF injection (Funk et al., 2009; Shchuko et al., 2015; Noma et al., 2016c; d; Matsushima et al., 2019) and parallel correlations between changes of aqueous VEGF concentration, visual acuity (VA), and optical coherence tomography (OCT) parameters after a single dose of intravitreal bevacizumab (IVB) (Noma et al., 2016c) or intravitreal ranibizumab (IVR) (Matsushima et al., 2019) injection were observed in RVO-ME patients.

Baseline intraocular VEGF levels may have value in predicting treatment response. Campochiaro et al. reported that baseline aqueous VEGF level was inversely correlated to VA improvement after 3 monthly IVR injections (Campochiaro et al., 2008). Similarly, Park, S.P. et al. detected higher baseline aqueous VEGF levels in patients who were unresponsive to a single IVB injection (Park and Ahn, 2009). However, in another study, Noma and others found that changes in aqueous VEGF after an IVB injection (1 month post-injection vs. baseline) were not associated with improvement of ME, although aqueous VEGF was suppressed to around the detection limit or lower in most patients (Noma et al., 2016d). The only study that measured aqueous VEGF at baseline and after corticosteroid treatment revealed that intravitreal dexamethasone implant (Ozurdex) has little effect on VEGF levels but causes a pan-suppression of aqueous inflammatory mediators (including interleukin (IL)-6, IL-8, monocyte chemoattractant protein -1 (MCP-1), soluble intercellular adhesion molecule-1 (sICAM-1), etc.) as well as angiopoietin (ANG)-2 levels (Rezar-Dreindl et al., 2017).

In other studies, baseline aqueous VEGF levels appeared less valuable than pro-inflammatory factors to predict ME recurrence. When Noma and others studied the correlation between aqueous factors and number of IVR injections needed to control ME recurrence during an observation period of 6 months, some pro-inflammatory cytokines (including IL-6, 8, etc.) but not VEGF were found to be correlated with the number of IVR injections (Noma et al., 2016a; Noma et al., 2021).

### IL-6

IL-6 is a key pro-inflammatory factor that can act on vascular endothelial cells and increase vascular permeability (Maruo et al., 1992; Alsaffar et al., 2018). Similar to VEGF, intraocular IL-6 was observed elevated in RVO eyes than normal controls (Campochiaro et al., 2009; Feng et al., 2013; Noma et al., 2014c; Jung et al., 2014; Sohn et al., 2014; Shchuko et al., 2015), and higher in major than macular BRVO (Lim, 2011; Noma et al., 2016b) and in ischemic than non-ischemic RVO (Noma et al., 2010b; Noma et al., 2010c; Funatsu et al., 2012; Koss et al., 2012; Noma et al., 2014a; Jung et al., 2014). It also positively correlated to central macular thickness (CMT) and/or nonperfusion area (NPA) (Noma et al., 2005; Noma et al., 2006; Noma et al., 2008; Noma et al., 2009, 2010b; Noma et al., 2010c; Kaneda et al., 2011; Noma et al., 2015), NVI (Chen et al., 1999; Ki et al., 2007), SRD (Noma et al., 2012b), and aqueous flare (Noma et al., 2013c; Noma et al., 2014b; Noma et al., 2017; Mashima et al., 2019). Indeed, significant correlations have been observed between VEGF and IL-6 in intraocular fluids of RVO (Noma et al., 2005; Noma et al., 2006; Ki et al., 2007; Noma et al., 2009; Noma et al., 2013c; Noma et al., 2015), and IL-6 was reported to be able to promote secretion of VEGF (Cohen et al., 1996; Noma et al., 2005).

Changes in intraocular IL-6 levels after intravitreal anti-VEGF treatment were conflicting. Funk et al. (2009) and Sohn, H.J. et al. (2014) reported that intraocular levels of cytokines and growth factors except VEGF were not significantly altered by IVB. However, Noma et al. showed a significant decrease in intraocular IL-6 level after the first IVB (Noma et al., 2016d), yet in another study of the same group, only borderline statistical significance was observed for change in IL-6 after 6 monthly IVB injections (Noma et al., 2016c). In studies with IVR, on the other hand, IL-6 levels were found to be significantly decreased after one dose of IVR (Mashima et al., 2019; Matsushima et al., 2019). The potential role of baseline IL-6 level for prediction of ME recurrence has been stated previously in the VEGF section, however, in another study by Campochiaro PA et al., no difference was observed in aqueous IL-6 levels between eyes with and without residual ME after two IVR injections (Campochiaro et al., 2009).

Decreases of intraocular IL-6 were also observed during intravitreal corticosteroid treatment, including intravitreal triamcinolone acetate (IVTA) (Sohn et al., 2014) and intravitreal dexamethasone implant (Querques et al., 2014; Rezar-Dreindl et al., 2017), and a decrease of aqueous IL-6 was found to be associated with improvement of ME (Rezar-Dreindl et al., 2017).

### IL-8

IL-8, also known as chemokine C-X-C motif ligand 8 (CXCL8), can be induced by injury and ischemia. It recruits neutrophils and other granulocytes and functions as a potent promoter of angiogenesis (Boisvert et al., 1998). IL-8 levels were found increased and positively correlated with the severity of ME and retinal ischemia in both BRVO (Noma et al., 2011d; Lee et al., 2012; Noma et al., 2014c) and CRVO (Noma et al., 2015), higher in CRVO than BRVO (Rezar-Dreindl et al., 2017), in major BRVO than macular BRVO (Lim, 2011; Noma et al., 2016b), and had a strong correlation with baseline aqueous flare value (Noma et al., 2017), NPA, CMT, as well as VA (Kaneda et al., 2011). Similar to IL-6, IL-8 was found to be able to stimulate the expression of VEGF in vascular endothelial cells (Martin et al., 2009). In addition to VEGF, correlations between intraocular IL-8 and MCP-1 levels were documented in different disease stages or scenarios, including at baseline (Noma et al., 2014c; 2015), during IVB treatment (Funk et al., 2009), post-treatment (Noma et al., 2016c), and in patients with insufficient efficacy (Shchuko et al., 2015).

Intraocular IL-8 level generally decrease along with absorption of ME in response to different treatment modalities including intravitreal anti-VEGF agents (Funk et al., 2009; Shchuko et al., 2015; Noma et al., 2016c; Noma et al., 2021), dexamethasone implant (Rezar-Dreindl et al., 2017) and vitrectomy (Okunuki et al., 2011). However, a single IVB, IVTA (Sohn et al., 2014; Noma et al., 2016d) or IVR (Matsushima et al., 2019) injection appeared insufficient to cause a statistically significant reduction of aqueous IL-8 level. Noma et al. revealed a significant reduction in aqueous IL-8 between the second and the third doses of IVB in both CRVO and BRVO eyes during a regimen of six monthly IVB injections, indicating a slow response of aqueous IL-8 downregulation (Noma et al., 2016c). Kotake et al. noted that two monthly injections of intravitreal aflibercept (IVA) significantly downregulated aqueous IL-8 but IVR did not, suggesting a stronger inhibitory effect of IVA than IVR on aqueous IL-8 (Kotake et al., 2019). Moreover, baseline IL-8 levels were found to be correlated with the number of IVR injections needed during a 6-month period with a “1 + PRN” regimen for BRVO (Noma et al., 2016a; Noma et al., 2021), which suggested the potential role of aqueous IL-8 as a predictor for ME recurrence.

### MCP -1

MCP-1 is a chemotactic cytokine also known as chemokine C-C motif ligand 2 (CCL2). It plays a critical role in monocyte recruitment (Ajuebor et al., 1998; Schober and Zernecke, 2007), and may participate in microvascular endothelial injury (Ajuebor et al., 1998; Hodge et al., 2012), which leads to the breakdown of the inner blood-retinal barrier in pathologic conditions (Klaassen et al., 2013). MCP-1 level was observed higher in eyes affected by RVO than control (Noma et al., 2011d; Noma et al., 2013b; Jung et al., 2014; Noma et al., 2014c; 2015), CRVO than BRVO (Jung et al., 2014; Noma et al., 2015), ischemic than non-ischemic RVO (Noma et al., 2014a; Jung et al., 2014; Noma et al., 2015), and positively correlated to CMT (Noma et al., 2011d; Noma et al., 2013b; Noma et al., 2014a; Noma et al., 2014c; 2015), NPA (Kaneda et al., 2011; Noma et al., 2013b), SRD thickness (SRT) (Noma et al., 2014c; 2015) and aqueous flare value (Noma et al., 2014b; Noma et al., 2017; Mashima et al., 2019), although some studies failed to found a significant relevance (Kunikata et al., 2012; Sohn et al., 2014).

Research has revealed a complex interplay between MCP-1 and other cytokines, the most notable of which is its synergistic effect with VEGF. VEGF can bind to VEGF receptor (VEGFR)-2 and enhance the expression of MCP-1 (and IL-8, sICAM-1, etc.) through nuclear factor-kappa B (NF-kB) (Ledebur and Parks, 1995; Baldwin, 1996; Marumo et al., 1997), while MCP-1 can recruit eosinophils that have been identified as an important source of VEGF (Horiuchi and Weller, 1997). In addition, the correlations between MCP-1 and IL-6 and IL-8 have also been documented (Noma et al., 2011d; Noma et al., 2014c; 2015).

Intravitreal injection of anti-VEGF agents (Funk et al., 2009; Shchuko et al., 2015; Noma et al., 2016a; Noma et al., 2016c; d; Matsushima et al., 2019; Noma et al., 2021) and corticosteroids (Kunikata et al., 2012; Rezar-Dreindl et al., 2017) generally leads to a significant decrease in intraocular MCP-1. While a significant association was found between the changes of aqueous MCP-1 and VEGF during IVB treatment (Funk et al., 2009), no statistical correlations between the reduction of MCP-1 and improvement in vision or ME were found in studies using IVR (Matsushima et al., 2019) or IVB (Noma et al., 2016d). During IVR treatment, although higher intraocular levels of IL-8 and MCP-1 were detected in patients with insufficient efficacy (Shchuko et al., 2015), no statistical relationship was found between the baseline MCP-1 level and the number of injections needed in a follow-up period of 6 months (Noma et al., 2016a; Noma et al., 2021). These contradictory findings suggest that more studies are needed before MCP-1 can be considered as an ideal biomarker for treatment response prediction and/or disease monitoring during intravitreal anti-VEGF treatments.

Notably, in a study on intravitreal dexamethasone implants, statistically significant correlations between decreases of MCP- 1, sICAM-1, ANG-2, and improvement of ME were found in both BRVO and CRVO, and the rise of intraocular MCP-1 was detected earlier than the recurrence of ME, suggesting a potential role of MCP-1 in disease monitoring during intravitreal corticosteroid treatment (Rezar-Dreindl et al., 2017).

### sICAM-1

sICAM-1 is a circulating form of ICAM-1 and both of them have been reported to be involved in the inflammatory processes of many diseases (Witkowska and Borawska, 2004). sICAM-1 concentration in intraocular fluids were significantly elevated as compared to control (Noma et al., 2011a; Noma et al., 2011b; Noma et al., 2011d; 2012d; Noma et al., 2013b; Noma et al., 2014a; Noma et al., 2014b; Noma et al., 2014c; 2015; Noma et al., 2016b), and have been found associated with signs indicative disease severity, including degree of retinal vascular involvement (macular BRVO vs. major BRVO) (Noma et al., 2012d; Noma et al., 2016b), aqueous flare value (Noma et al., 2017; Mashima et al., 2019), degree of retinal ischemia (Noma et al., 2011a; Noma et al., 2011d; c; Noma et al., 2013c; Noma et al., 2014b; Noma et al., 2014c), CMT (Noma et al., 2013b; Noma et al., 2014c), and SRT (Noma et al., 2011d; Noma et al., 2014c).

Unlike MCP-1, intraocular sICAM-1 was not significantly suppressed by intravitreal anti-VEGF agents (Noma et al., 2016d; c; Mashima et al., 2019; Matsushima et al., 2019; Noma et al., 2021), and no significant correlations were found between changes in aqueous sICAM-1 level and improvements in visual acuity, ME (Noma et al., 2016d), or aqueous flare (Mashima et al., 2019). On the contrary, a significant decrease of aqueous sICAM-1 was observed after IVTA (Noma et al., 2013a).

Baseline sICAM-1 levels may have predictive value for disease recurrence. It was reported to be associated with aqueous flare values at first recurrence (Noma et al., 2017) as well as the number of IVR injections needed during a period of 6 months (Noma et al., 2016a; Noma et al., 2021).

### IL-12 and IL-13

IL-12 is a key pro-inflammatory cytokine that drives the induction of naive CD4^+^ T lymphocytes into Th1 cells and activation of other immune cells such as neutral killer cells (Trinchieri, 1995). IL-13 is an inducer of Th2-type cytokines and plays an important role in the pathogenesis of allergy, cancer, and tissue fibrosis (Karam et al., 2011; Van Dyken and Locksley, 2013). Intraocular IL-12 and IL-13 were generally reported to be elevated in RVO (Noma et al., 2011d; Kaneda et al., 2011; Noma et al., 2014c, 2015; Shchuko et al., 2015; Noma et al., 2016b), although in one study they were not significantly different between BRVO and cataract eyes (Lee et al., 2012), and another study showed that IL-12 was even significantly lower in CRVO than cataract eyes (Rezar-Dreindl et al., 2017).

Correlations between intraocular levels of IL-12 and IL-13 in RVO eyes have been observed (Noma et al., 2011d; Noma et al., 2014c; 2015). While they were demonstrated to be negatively correlated to retinal ischemia, CMT and SRT in several studies (Noma et al., 2011d; Noma et al., 2014c), they were reported to not be significantly correlated with aqueous flare value (Noma et al., 2017; Mashima et al., 2019) and were not higher in major BRVO than macular BRVO (Noma et al., 2016b).

In BRVO, intraocular IL-12 and IL-13 levels were reported to not have significantly changed after IVB (Noma et al., 2016d) or IVR injections (Noma et al., 2021). However, in a study that observed the kinetics of multiple cytokines during a regimen of 6 monthly IVB injections, aqueous IL-13 was significantly suppressed after 3 consecutive IVB injections in BRVO but remained unchanged during the regimen in CRVO; and aqueous IL-12 remained changed in BRVO but significantly increased after 3 IVB injections in CRVO (Noma et al., 2016c). They proposed a protective anti-inflammatory effect of IL-12 and a pathogenetic pro-inflammatory role of IL-13 in RVO. The different responses of aqueous IL-12 and IL-13 levels after repeated IVB injections between CRVO and BRVO could be explained by the different extent of ocular damage involved between these two RVO subtypes (Noma et al., 2016c).

It is uncertain whether intraocular IL-12 or IL-13 levels have predictive value for treatment response. Kaneda et al. (2011) revealed a significant association between IL-12 level and refractoriness to IVB in BRVO, and Shchuko et al. (2015) presented a higher level of IL-13 in RVO patients with insufficient response to IVR. However, Noma et al. found no correlation between intraocular IL-12 or IL-13 levels and the number of IVR injections needed during a 6-month follow up period with a 1 + PRN regimen (Noma et al., 2016a).

### sVEGFR-1 and sVEGFR-2

Soluble VEGF receptors (sVEGFR)-1 and sVEGFR-2 are soluble forms of VEGF receptors (Ebos et al., 2008). sVEGFR-1, a receptor for VEGF, VEGF-B and PlGF, is a pro-inflammatory factor (Clauss et al., 1996; Kiba et al., 2003; Murakami et al., 2006), and sVEGFR-2 was reported to have anti-angiogenic activity (Maynard et al., 2003; Jacobi et al., 2004; Ebos et al., 2008) and promote vascular maturation by mediating the interaction between endothelial cells and mural cells (Lorquet et al., 2010).

sVEGFR-1 appeared to be a promising candidate biomarker for RVO. Activation of sVEGFR-1 by its ligands leads to the production of pro-inflammatory and pro-angiogenic mediators by macrophages and microglia in the retina (Ziche et al., 1997; Crespo-Garcia et al., 2017; Uemura et al., 2021). sVEGFR-1 was reported higher in intraocular fluids of RVO than control (Noma et al., 2011d; Noma et al., 2014c; 2015; Noma et al., 2016b), higher in ischemic than non-ischemic CRVO (Noma et al., 2015), and significantly decreased in response to anti-VEGF treatments (Noma et al., 2016d; c; Matsushima et al., 2019; Noma et al., 2021). In addition, intraocular sVEGFR-1 level significantly correlated with flare value at both baselines and recurrences (Noma et al., 2017; Mashima et al., 2019), and correlated with the number of injections needed during a 6-month “1 + PRN” IVR regimen (Noma et al., 2016a; Noma et al., 2021).

The intraocular level of sVEGFR-2 was also reported to be elevated in RVO (Noma et al., 2011b; Noma et al., 2011d; Noma and Mimura, 2013; Noma et al., 2013b; Noma et al., 2014b; Noma et al., 2014c; Noma et al., 2016b), however, its relationship with disease severity and refractoriness was less conclusive. Intraocular sVEGFR-2 was observed to be correlated with ME and SRT (Noma et al., 2011d; Noma et al., 2013b; Noma et al., 2014b; Noma et al., 2014c), but not with aqueous flare value (Noma et al., 2017; Mashima et al., 2019) and ischemic status in CRVO (Noma et al., 2015). In addition, no significant difference between intraocular sVEGFR-2 levels at baseline and 1 month after IVB (Noma et al., 2016d; c) was observed, and the correlations between baseline aqueous sVEGFR-2 level and the number of IVR injections needed within 6 months of a “1 + PRN” IVR regimen were conflicting in two studies (Noma et al., 2016a; Noma et al., 2021).

### PDGF-AA

Platelet-derived growth factor (PDGF) is a growth factor that regulates the migration of mesenchymal cells (Hossain et al., 1998; Mamer et al., 2017) and has been reported to have a role in ocular neovascularization induced by hypoxia (Benjamin et al., 1998; Clapp et al., 2009). Intraocular PDGF-AA (an isoform of PDGF) level was reported higher in RVO than control (Noma et al., 2011d; Lee et al., 2012; Noma et al., 2014c, 2015; Jung et al., 2014; Noma et al., 2016b), in CRVO than BRVO (Jung et al., 2014), in major BRVO than macular BRVO (Lim, 2011; Noma et al., 2016b), and positively correlated to retinal ischemia, ME, and SRT in BRVO (Noma et al., 2011d; Noma et al., 2014c). However, exceptional results were derived in some studies that showed intraocular PDGF-AA was not elevated in RVO as compared to normal control (Lim, 2011; Sohn et al., 2014; Rezar-Dreindl et al., 2017). PDGF-AA was significantly correlated to aqueous flare values at baseline, in one study (Mashima et al., 2019) but not in another (Noma et al., 2017).

The aqueous humor level of PDGF-AA decreased significantly over time after multiple IVB (Noma et al., 2016c) or IVR injections (Noma et al., 2021). Notably, baseline aqueous PDGF-AA level was associated with the number of IVR injections needed in a period of 6 months during a “1 + PRN” regimen (Noma et al., 2016a; Noma et al., 2021), suggesting the potential role of aqueous PDGF-AA in the prediction of ME recurrence.

### Other Biomolecules

In addition to the biomolecules mentioned above, many other molecules have also been studied in RVO (Table 3), among which the following were measured in more studies than others, and thus are discussed briefly below, including pigment epithelium-derived factor (PEDF), PlGF, tumor necrosis factor (TNF)-α, erythropoietin (EPO), pentraxin 3 (PTX3), and nitric oxide (NO).

PEDF is a cytokine that has anti-angiogenic properties (Rychli et al., 2009) and was observed to antagonize the effect of VEGF in retinal neovascularization (Mori et al., 2001; Duh et al., 2002). Intraocular PEDF level was lower in both BRVO and CRVO as compared to control and was observed negatively correlated to retinal thickness (Noma et al., 2010a).

PlGF belongs to one of the VEGF subfamilies and is also a pro-angiogenic factor (Shibuya, 2008). In RVO, aqueous PlGF was reported positively correlated CMT, aqueous flare value and severity of ischemia (Noma et al., 2011d; Noma et al., 2017).

TNF-α is a key inflammatory cytokine which has pleiotropic effects on various cells and plays an important role in inflammation, cell proliferation and apoptosis. It can increase the permeability of vascular endothelium (Chen and Goeddel, 2002) and may participate in the pathogenesis of ocular inflammation, edema, and neovascularization (Rodrigues et al., 2009). TNF-α was increased in the intraocular fluids of RVO with higher levels found in ischemic compared to non-ischemic RVO (Jung et al., 2014; Zeng et al., 2019).

EPO, a hormone produced in kidney and fetal liver that regulates erythropoiesis, has pleiotropic functions including antioxidant, angiogenic, and neuroprotective activities (Junk et al., 2002; Watanabe et al., 2005). Upregulation of vitreous EPO was observed in both BRVO and CRVO, with higher levels detected in more ischemic subjects (Inomata et al., 2004; Stahl et al., 2010; Shin et al., 2014). These findings are explained by the fact that EPO production is primarily stimulated by hypoxia (Inomata et al., 2004; Weidemann and Johnson, 2009).

PTX3, a member of the acute response protein family Pentraxin, is associated with vascular injury (Peri et al., 2000; Suzuki et al., 2008), and has been proposed as a prognostic biomarker of myocardial infarction and heart failure (Latini et al., 2004; Suzuki et al., 2008). Interestingly, intraocular PTX3 was reported significantly increased in RVO patients with more profound changes observed in ischemic subtype (Noma et al., 2013b; Noma et al., 2014a), and reduced in response to dexamethasone implant (Campochiaro et al., 2015).

NO is a free radical gas molecule synthesized by nitric oxide synthase (NOS). It has a vasodilatory effect, which increases blood flow and is beneficial for vascular occlusive conditions; however, it can be neurotoxic when generated in excess (Dawson et al., 1994; Donati et al., 1998; Sennlaub et al., 2002). Aqueous humor NO levels were found significantly higher in RVO than control (Fard and Dehpour, 2010; Fard et al., 2010), but further studies are needed to unravel its role in disease pathogenesis of RVO.

While evidence is accumulating that PEDF, PlGF, TNF-α, EPO, PTX3, and NO may be involved in the pathogenesis of RVO and may be associated with disease severity, their roles as predictive factors for treatment response are less well studied.

## Discussion and Conclusion

**FIGURE 1:**
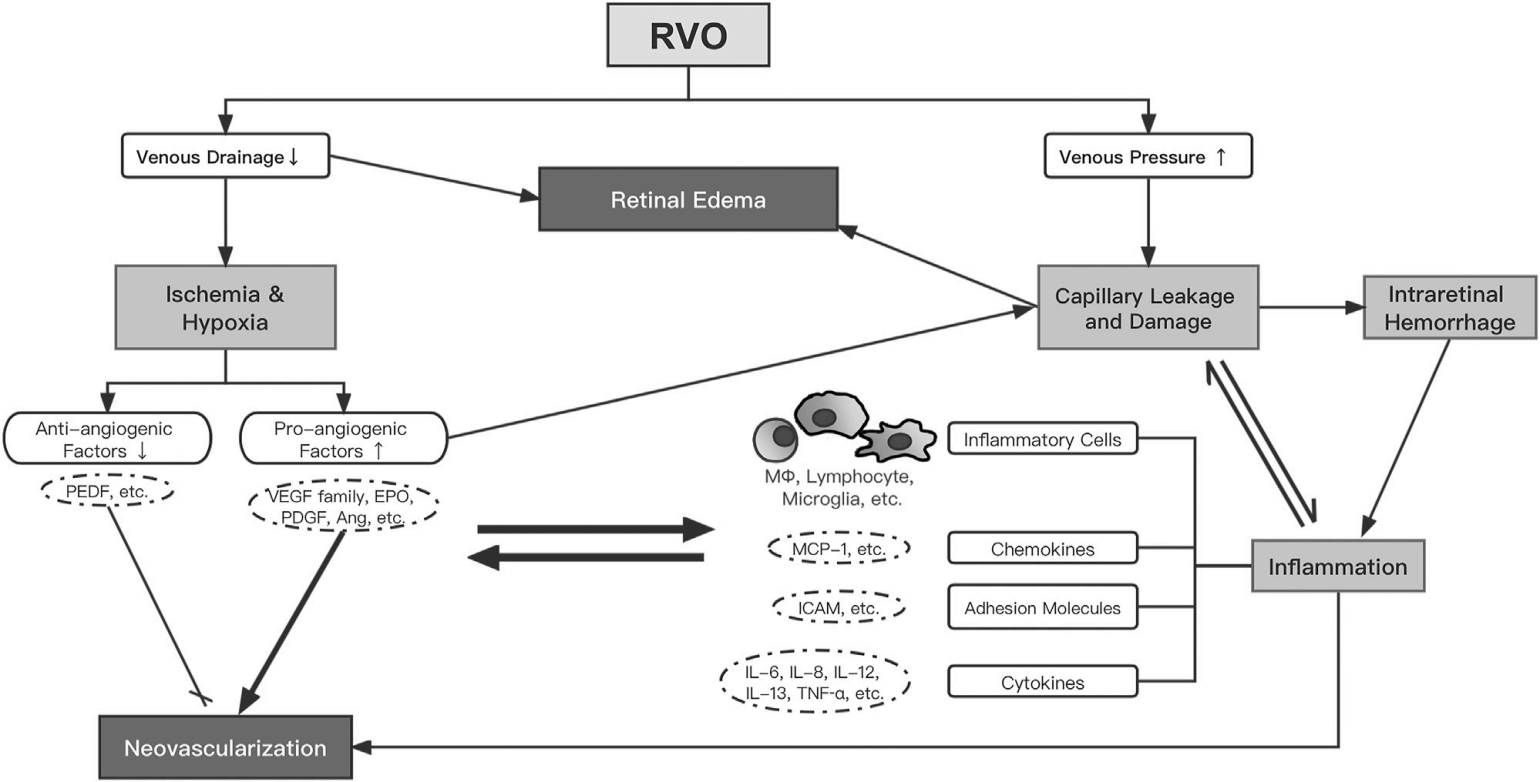
The proposed network of pathogenesis in RVO. Abbreviations: RVO, retinal vein occlusion; VEGF, vascular endothelial growth factor; EPO, erythropoietin; PDGF, platelet-derived growth factor; Ang, angiopoietin; PEDF, pigment epithelium-derived factor; MΦ, macrophage; ICAM, intercellular adhesion molecule; IL, interleukin; MCP, monocyte chemoattractant protein; TNF, tumor necrosis factor.

Previous studies have investigated a wide range of biomolecules in the aqueous or vitreous of RVO eyes with various clinical characteristics or at different clinical stages and have generally delineated the complex network of pathogenetic mechanisms. Impaired blood drainage and increased venous pressure following RVO result in ischemia/hypoxia and capillary leakage/damage, which upregulate pro-angiogenic factors (VEGF, PlGF, VEGFR, EPO, PDGF, ANG, etc.) and cause inflammation. Retinal edema develops as a result of reduced venous drainage and increased capillary leakage/permeability, which is augmented by a variety of pro-angiogenic and inflammatory mediators (Figure 1).

Of note are the pleiotropy of the cytokines and the complex synergistic cross-talk among them. VEGF, the major pro-angiogenic factor, has strong stimulative effects on migration and proliferation of endothelial cells, but it also impedes pericyte recruitment by disrupting PDGF-related pathways *via* VEGF-R2-mediated signaling (Ferrara et al., 2003; Greenberg et al., 2008), which impairs vessel maturation and increases vascular leakage. VEGF also enhances vascular permeability by altering endothelial cell tight junctions (Vinores et al., 1999), in which NO-related signals may be involved (Lakshminarayanan et al., 2000). In addition, VEGF interacts with inflammatory mediators through NF-kB associated pathways, including IL-6, IL-8, IL-12, TNF-α, and MCP-1 (Kulms and Schwarz, 2006). These inflammatory mediators may affect vascular permeability through VEGF-dependent (Martin et al., 2009) and -independent mechanisms, such as IL-8, which can directly downregulate tight junctions (Yu et al., 2013). The sources of these cytokines are multiple, involving not only inflammatory cells but also a variety of ocular resident cells. For example, IL-6 was found to be secreted by Müller cells (Liu et al., 2015), endothelial cells (Akira et al., 1993) and retinal pigment epithelial (RPE) cells (Elner et al., 1992).

The synergism between VEGF and inflammation in RVO pathogenesis is also in line with the fact that both anti-VEGF treatments and corticosteroids are effective for the majority of RVO-ME patients. Anti-VEGF agents suppress intraocular VEGF to a sub-physiological level, resulting in a variety of anti-inflammatory effects, whereas intravitreal corticosteroids cause pan-suppression of inflammatory mediators by affecting VEGF-related downstream signals (Edelman et al., 2005; McAllister et al., 2009) and turning off activated inflammatory genes (Barnes, 2006). The varying results on intraocular biomolecule changes after intravitreal anti-VEGF or corticosteroid treatments among studies may be due to the complexity of disease pathogenesis, inter-individual heterogeneity of disease phenotype, differences in study design, and biomolecular analysis methods.

Current studies have demonstrated that intraocular levels of pro-angiogenic and inflammatory mediators can reflect disease phenotype, severity, treatment response, and/or refractoriness, providing the basis for using these molecules as candidates for companion diagnostic biomarkers. Based on the current review, a panel of biomolecules, which includes VEGF, IL-6, IL-8, MCP-1, sVEGFR-1, sICAM-1, and PDGF-AA, may be valuable to assess the involved mechanisms and/or disease severity at baseline; VEGF might be valuable to predict the response of the first intravitreal anti-VEGF treatment; IL-6, IL-8, MCP-1, sICAM-1, and PDGF-AA may provide valuable information on refractoriness or middle-term (6 months) requirements of anti-VEGF injections. On the other hand, however, there is a long way ahead to configure a preliminary panel of biomarkers for intravitreal corticosteroids as well as for anti-VEGF agents.

## Clinical Perspectives and Application

Aqueous humor appeared to be adequate as a source of intraocular biomarkers. In a meta-analysis involving 116 studies, authors revealed that significant differences in levels of most intraocular cytokines between RVO and control can be observed in both the vitreous and aqueous humor, including VEGF, IL-6, IL-8, and MCP-1 (Minaker et al., 2020). The current techniques of aqueous humor collection using small gauge syringes, however, are not safe and convenient enough to be performed routinely in an outpatient clinic. The novel disposable aqueous humor collector developed by our group, which facilitates one-handed anterior chamber paracentesis and accurate aqueous sampling, may overcome these limitations in the future (Qu et al., 2020). The most advanced multiplex immunoassay platforms, which allow simultaneous analysis of multiple biomarkers with a minimum sample volume requirement, have laid the technical basis for the development of aqueous humor-based companion diagnostics for ocular diseases.

Despite the significant advances achieved to date, more effort is needed to narrow down the range of possible biomarkers, to develop test kits specifically for particular companion diagnostic purposes, and to validate these kits in well-designed clinical trials. Ultimately, for widespread acceptance in the ophthalmic community and patients, a non-invasive assay of these biomarkers would be most useful.
